# Letter to the Journal

**Published:** 1878-12

**Authors:** 


The following communication published, *verbatim et literatim*, requires no explanation. We suggest that the author be invited to participate in the proceedings of the Buffalo Medical Association, and possibly "*read a paper.*"

Mr. Editor & Proprietor I am not in sport or speaking foolish let there Be a Improvement in surgery and medical science, and the only way is to go about it Right experiment and find out (as a matter of Charity Philathanopy to wake up the medical fraternity, Pleas Present it to your Readers (the man who Can accomplish these things skillfully has found out a scientific fact and will make money for himself N, B if there is no such a Paper Published in Buffalo, why whoevers hands this falls into, Pleas send it to some other Surgical Paper Published Published here is stamped Enveope to do so so it cost you nothing to do so Pleas do it.

Nov 18,78

Wooster O in Sincerety Present this to your medical Readers everywhere

To Pleas Present this to your, ☜ numerous surgical & Medical Subscribers, one of the first and greatest medical Experiments of our Surgeons, our Day ought to Be engrafting animal Tissues or ☜ membranes in the human Body for Instance where a man had lost a Testicle=take the Testicle of a Bull or Boar or stallion, attach it to him, get the Blood to Circulate freely through the Parts freely & apply Elecricty & Prevent slooghing and mortification & get the Parts to grow together, I beleive it Can Be done successfully, let the experiment Be tried Perseverelingly on the lower animals first, then on man, yes sir the surgeon who Can suceed will stand at the head of his Profesion, so likewise in other malidies of the Body let these experiments Be tried ☞ there is very few Important Inventions & Cures & Instruments & Discoveries Been made, without first experimenting and making great effort at first to accomplish the desired end in view yours truly G- R- W

so Pleas Present free gratis to your Readers a suffer

P- S we have Plenty of Books treating on Seminal weakness =But I Cannot=find=any treating on them=other attending Diseases of which I Beleive thousands are suffering with vis orchitis & Neuralgia Hydrocle & Tumors & Hardening falling loosing of the Cord & Spermatocle Ruptured artery & varicose veins abcesses & scrofula sore & variocle & Cancer & Cartlige and hardning of the testicles & fungus Protusions Atrophy and the hundred and one other Diseases affecting the Testicles, why is it we have no Books on these very Important Diseases, a Book Costing two or three Dollars, with numerous engraving Pointing out on what Part of the testicle the Diseases Commences. the Causes that produces them ☞ also the way to Cure them to a healthy Condition=Both=By=Surgery=and=medicine I think would Be accepteable to thousands of suffers, the most of our Doctors know But very little about these Diseases, hardly know what to Call it, and Can scarcly ever Cure a sufferer.

